# Comparison of left ventricular geometry after HeartMate II and HeartWare left ventricular assist device implantation

**DOI:** 10.1186/1749-8090-8-31

**Published:** 2013-02-28

**Authors:** Morteza Tavakkoli Hosseini, Aron Frederik Popov, Andre Ruediger Simon, Mohamed Amrani, Toufan Bahrami

**Affiliations:** 1 Department of Cardiothoracic Surgery and Transplantation Harefield Hospital, Harefield, London, UK; 2 Department of Thoracic Cardiovascular Surgery, University of Göttingen, Göttingen, Germany

**Keywords:** Cardiomyopathy, Heart failure, Ventricular assist device, Echocardiography

## Abstract

**Background:**

HeartMate II (HM II) and HeartWare (HW) Left Ventricular Assist Devices have been successfully used in end-stage heart failure patients as a bridge to transplantation, recovery, or decision. We set out to compare their effect in off-loading the left ventricle and its geometry.

**Methods:**

The left ventricular end diastolic (LVEDD) and end systolic (LVESD) diameters were compared between first time HM II (n = 25) and HW implantations (n = 24) before and after the operation at 1, 3, and 6 months. A p value of less than 0.05 was considered as significant.

**Results:**

Post-operative LVEDD and LVESD at 1, 3, and 6 months were significantly reduced in comparison with pre-operative values in both HM II and HW groups. No significant difference was found comparing HM II and HW groups together before and after the operation.

**Conclusions:**

Our study shows that both HM II and HW can significantly reduce the left ventricular systolic and diastolic dimensions and off-load the left ventricle. The miniaturized nature of HW does not affect its performance and it could be as effective as HM II.

## Background

Mechanical circulatory support for patients with advanced heart failure has evolved considerably during the past 30 years and is now the standard therapy at many medical centers worldwide [1]. Ventricular Assist Devices (VAD) have been mainly used as a bridge to transplantation. Nowadays, as a result of donor shortage and the success of the newer devices, they are also considered as a bridge to recovery or a bridge to decision [2].

In the last decade, the smaller second generation continuous-flow assist devices have successfully replaced their first generation pulsatile predecessors. They have proved to be more reliable, smaller in size, and have lower rates of adverse events (infection, bleeding, stroke, and device failure). The most common second generation devices currently used are HeartMate II (Thortec Inc.) [3], Micromed Debakey [4], Berlin Heart Incor (Berlin Heart AG) [5], and Jarvik 2000 (Jarvik Heart Inc.) [6]. HeartMate II is the most successful and widely used second generation VAD.

Another recent addition to this family is HeartWare VAD (HeartWare Inc). It is a miniaturized centrifugal blood pump that could be placed intra-pericardially, thereby avoiding abdominal surgery and pump pocket formation. It has proved to provide satisfactory long term survival with excellent quality of life, low rate of adverse events and feasibility for implantation via minimally invasive incisions [7-10]. There has been a debate if the miniaturized nature of the pump could affect its effect on off-loading of the left ventricle, in comparison with the bigger second generation VAD systems. We set out to compare the effect of HeartWare (HW) and HeartMate II (HM II) VAD systems in off-loading the left ventricle and its geometry.

## Methods

First time HM II and HW implantations for Dilated Cardio-Myopathy performed at Harefield Hospital from April 2008 to October 2010 were included in this study. Pre-operative and post-operative (first month and then three-monthly) echocardiography is performed at Harefield Hospital as a routine follow up procedure for all patients with a VAD implantation. Echocardiography data is inclusive of the left ventricular systolic diameter and the left ventricular diastolic diameter at baseline VAD pump speed (9600 rpm for HM II and 2800 rpm for HW).

This data was part of Harefield Hospital post VAD surgery data collection and outcome evaluation. The study was categorized as a Service Evaluation by the Ethical Committee and required no need for ethical approval.

Fisher’s exact test was used for comparing the demographic factors, and the mortality. Pre-operative left ventricular parameters were compared with the post-operative left ventricular parameters at 1, 3, and 6 months after the operation, using Analysis of Variance (ANOVA) test. A p value of less than 0.05 was considered as significant. The study design was a retrospective review of the prospectively collected data.

## Results

HM II and HW groups contained 25 and 24 patients respectively. All patients had a history of Dilated Cardio-Myopathy with NYHA class 4 symptoms. Table 1 shows the demographics of the two groups. The mean age in HW group (50.29) was significantly higher than the mean age in HM II group (41.32). Six months mortality in HM II and HW groups were 9 (36%) and 4 (16%) respectively, with no significant difference. Table 2 shows the systolic and diastolic left ventricular diameters before and after VAD implantation. There was a significant difference between pre-operative and post-operative left ventricular diameters in each group. No significant difference was noticed in post-operative left ventricular diameters of each group. When comparing HM II and HW groups together, there was no significant difference in the pre-operative or post-operative left ventricular diameters in between the two groups.

**Table 1:** Pre-operative patients’ demographics

	**Number**	**Male**	**Female**	**Age**
HeartMate II	25	19 (76%)	6 (24%)	41.32 +/− 12.29
HeartWare	24	19 (79%)	5 (21%)	50.29 +/− 11.91

**Table 2:** Pre and post VAD implantation left ventricular diameters

	**Pre operative**	**1 Month**	**3 Months**	**6 Months**
HM II LVEDD mm	72.8 +/− 9.3	54.7 +/− 16.7	54.1 +/− 14.5	56.5 +/− 13.7
HW LVEDD mm	68.9 +/− 10.1	57.5 +/− 10.4	57.9 +/− 11.2	59.3 +/− 8.2
HM II LVEDD%	***	−25.1 +/− 21.0	−27.3 +/− 17.1	−24.7 +/− 15.4
HW LVEDD%	***	−17.3 +/− 13.0	−17.7 +/− 16.5	−14.9 +/− 12.3
HM II LVESD mm	66.0 +/− 9.3	47.0 +/− 15.8	44.5 +/− 16.2	45.9 +/− 15.3
HW LVESD mm	59.8 +/− 10.7	51.1 +/− 12.5	50.1 +/− 11.7	50.6 +/− 8.5
HM II LVESD%	***	−28.4 +/− 21.9	−34.2 +/− 20.0	−32.9 +/− 18.9
HW LVESD%	***	−17.0 +/− 17.1	−19.5 +/− 19.7	−18.3 +/− 15.2

***HM II***: HeartMate II; ***HW***: HeartWare; ***LVEDD***: Left Ventricular End Diastolic Diameter; ***LVESD***: Left Ventricular End Systolic Diameter; ***LVEDD*%**: Left Ventricular End Diastolic Diameter change compared to each patient’s pre-operative value; ***LVESD*%**: Left Ventricular End Systolic Diameter change compared to each patient’s pre-operative value.

## Discussion

Ventricular Assist Device systems have revolutionized the management of end stage heart failure. They are used as a bridge to transplantation, recovery, or decision [1,2,7-9,11]. There is a growing trend towards the use of smaller and more compact devices, with the aim of avoiding abdominal surgery, pump pocket formation, and reduction of the adverse events. Smaller devices also provide the chance for minimally invasive incisions, instead of a conventional median sternotomy [10]. HM II is the most successful and widely used second generation VAD system [1-3]. Echocardiographic studies show that HM II reduces the left ventricular end-diastolic dimension by 21% and 35%, one week and 4 months after VAD implantation respectively [12]. It has been quoted that patients with a relatively small left ventricular end diastolic diameters (<63 mm) have a significantly higher risk for in-hospital mortality [13]. Maybaum et al. reported in their study for cardiac recovery that HeartMate, Novacor, and Debakey VAD systems significantly decrease the left ventricular end diastolic diameters at 1, 2, 3, and 4 months after VAD implantation [14]. In the initial clinical experience with HW, no comparison was made between the pre-operative and post-operative left ventricular dimensions [7,8]. Our study confirms that HW significantly reduces the left ventricular systolic and diastolic dimensions and off-load the left ventricle in the short and long term. The miniaturized nature of the pump does not affect its performance, as no significant difference was noticed while comparing HW and HM II. We did not checked for the left ventricular dimensions at partial pump support/pump off status, as the aim of the study was not to check for myocardial recovery. Myocardial recovery is a debatable discussion in the current era with so many proponents and opponents [11,14]. Groups were not sub-analyzed based on the etiology of Dilated Cardio-Myopathy. Ethiology affects the outcome and the potential for recovery, but not the mechanical ability of the device in off-loading the left ventricle. So far, the implantation of smaller VAD systems via median sternotomy or minimally invasive incisions, has proved to be successful with satsifactory clinical and echocardiographic results [7,8,10].

## Conclusions

Our study shows that both HM II and HW can significantly reduce the left ventricular systolic and diastolic dimensions and off-load the left ventricle. The miniaturized nature of HW does not affect its performance and it could be as effective as HM II.

## Competing interest

The authors declare that they have no competing interests.

## Authors’ contributions

MTH and AFP designed the study, collected the data, wrote and revised the manuscript. MA, and ARS carried out the surgical procedures, and revised the manuscript. TB carried out the surgical procedures, co-wrote and revised the manuscript, supervised and lead the study. All authors read and approved the final manuscript.
